# Supplementary material for: Pilot Study of Heat-Stabilized Rice Bran Acceptability in Households of Rural Southwest Guatemala and Estimates of Fiber, Protein, and Micro-Nutrient Intakes among Mothers and Children
Source: Nutrients. 2024 Feb 5;16(3):460. doi: 10.3390/nu16030460 (PMC10856929; doi:10.3390/nu16030460)
Supplement: Supplementary file 1 [file nutrients-16-00460-s001.zip › Supplemental figures.pdf]

riceBran technologies™

nutrition by nature®

Typical Nutritional Data
RiBran 300

Typical Nutritional Data

Macronutrients	
Proteins (g / 100g) (AOAC 991.20.I)	14
Fat (g / 100g) (AOAC 960.39)	21
Saturated Fatty Acids (g / 100g) (AOCS Ce 1e-91)	4
Total Carbohydrate (g / 100g) (Calculation)	49
Available Carbohydrate (g / 100g) (Calculation)	24
Ash (g / 100g) (AOAC 945.46)	10
Moisture (g / 100g) (AACC 44-40)	6
Total Dietary Fiber (g / 100g) (AOAC 991.43)	25
Soluble Fiber (g / 100g) (AOAC 991.43)	2
Energy (kcal / 100g) (Calculation)	330.5

Minerals	
Sodium (mg / 100g) (ICP) (AOAC 984.27)	8
Potassium (mg / 100g) (ICP) (AOAC 984.27)	1570
Calcium (mg / 100g) (ICP) (AOAC 984.27)	40
Magnesium (mg / 100g) (ICP) (AOAC 984.27)	730
Phosphorus (mg / 100g) (ICP) (AOAC 984.27)	1590
Iron (mg / 100g) (ICP) (AOAC 984.27)	8

Phytosterols	
Beta Sitosterol (mg / 100g) (HPLC) (FAO JECFA)	167
Stigmasterol (mg / 100g) (HPLC) (FAO JECFA)	63
Campesterol (mg / 100g) (HPLC) (FAO JECFA)	96
Other Sterols (mg / 100g) (HPLC) (FAO JECFA)	15
**Total Phytosterols** (mg / 100g) (HPLC) (FAO JECFA)	**340**

Micronutrients	
Choline (mg / 100g) (J Lipid Res 26-1985)	105
Inositol (mg / 100g) (Food Chem 87-2004)	1500
gamma-Oryzanol (mg / 100g) (in house) (UV-Vis)	245
**Vitamins**	
**Carotenoids**	
Beta carotene (iu / 100g) (CIFSTJ 1982 15:16)	ND
Lutein (mcg / 100g) (in house) (HPLC H114)	100.0
Zeaxanthin (mcg / 100g) (USP / NF)	39.0
**Vitamin B Complex** (mg / 100g)	
Vitamin B1 (Thiamine) (AOAC 942.23)	2.7
Vitamin B2 (Riboflavin) (AOAC 970.65)	0.3
Vitamin B3 (Niacin) (AOAC 44.13)	47.0
**Vitamin E Complex** (HPLC) (AACC 86-06)	
Tocopherols (ppm)	12
Tocotrienols (ppm) (sum of alpha beta gamma delta)	13
**Total Sugars** (g / 100g) (AOAC 980.13)	**8.1**

Other Analysis	
Appearance (in house)	Extra Fine Granule
Color (in house)	Tan
Flavor (in house)	Nutty
Bulk Density (ASTMD 1895B, + / – 10%)	~0.49 g/cm3
Particle Size Distribution (laser scattering analyzer)	Minimum
through 850 micron (20 mesh)	95%
through 425 micron (40 mesh)	90%
through 250 micron (60 mesh)	70%
through 180 micron (80 mesh)	55%
through 150 micron (100 mesh)	50%

We will assume no legal responsibility due to inaccuracy, incompleteness or other errors or omissions contained in this document. In no event shall the company be liable for any claims losses, or damages due to such errors or omissions contained herein. The content of this document is subject to change. Please contact us for the latest version of this document or for further information. Since the user's product formulations, specific use applications and conditions of use are beyond our control, we make no warranty or representation regarding the results which may be obtained by the user. It is the responsibility of the user to determine the suitability of our products for the user's specific purposes and the legal status for the user's intended use of our products.

RiBran 300 TNDS, Rev. 3
Supersedes all previous versions

Created: February 10, 2016
Effective: January 1, 2016
Expires: January 1, 2017

6720 N. Scottsdale Road, Suite 390 • Scottsdale, AZ 85253 • Phone: 602.522.3000 • Fax: 602.522.3001 • www.RiceBranTech.com

Supplemental Figure S1. Spec sheet for the heat-stabilized rice bran (RiBran 300) used in this study.

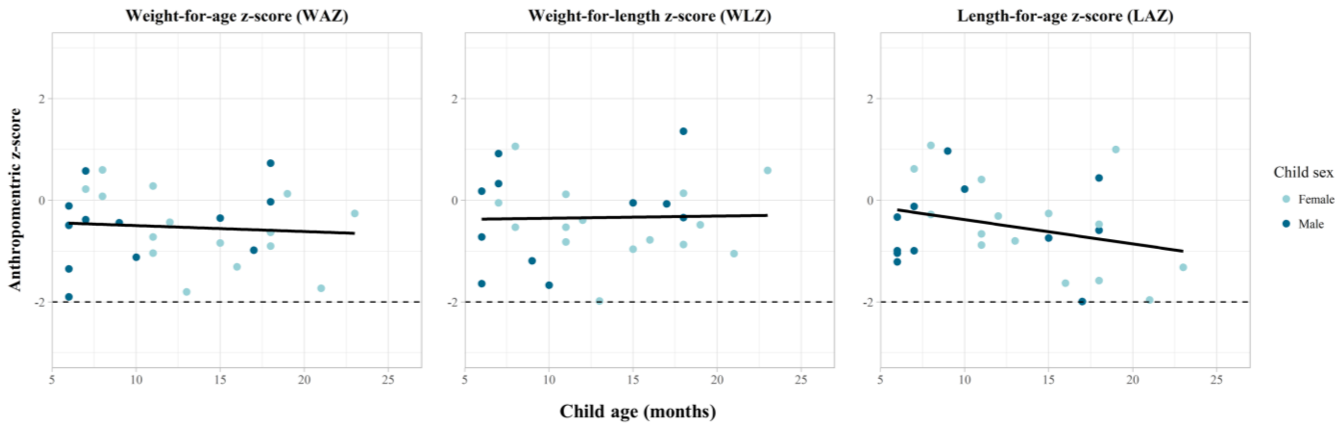

Supplemental Figure S2. Child (n=27) baseline anthropometric z-scores for weight-for-age (WAZ), weight-for-length (WLZ), and length-for-age (LAZ). Scatter plots show the three z-score measures based on child baseline age in months and by sex (female/male).

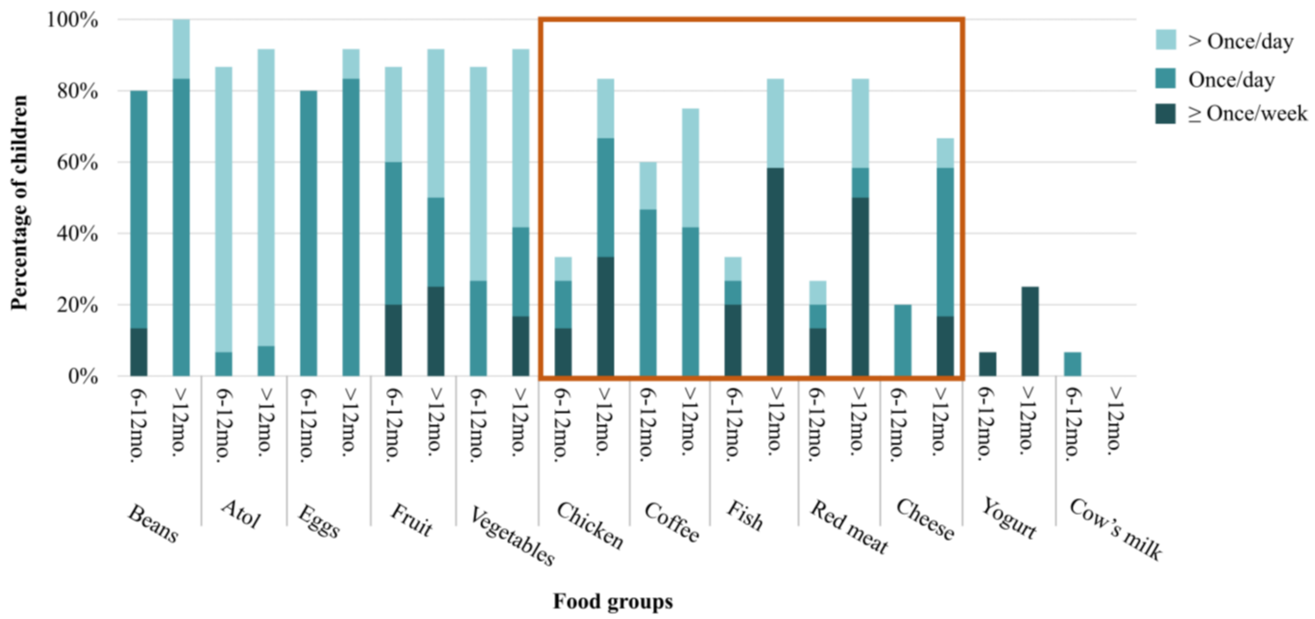

Supplemental figure S3. Healthy child (n=27) baseline food frequency questionnaire (FFQ) results for 12 of the 17 food groups included in the FFQ and split by child age (6-12 months / >12 months). mo. = months. Bars are colored by percentage of children consuming each food group at least once per week (darkest color), once per day (middle color), and more than once per day (lightest color).

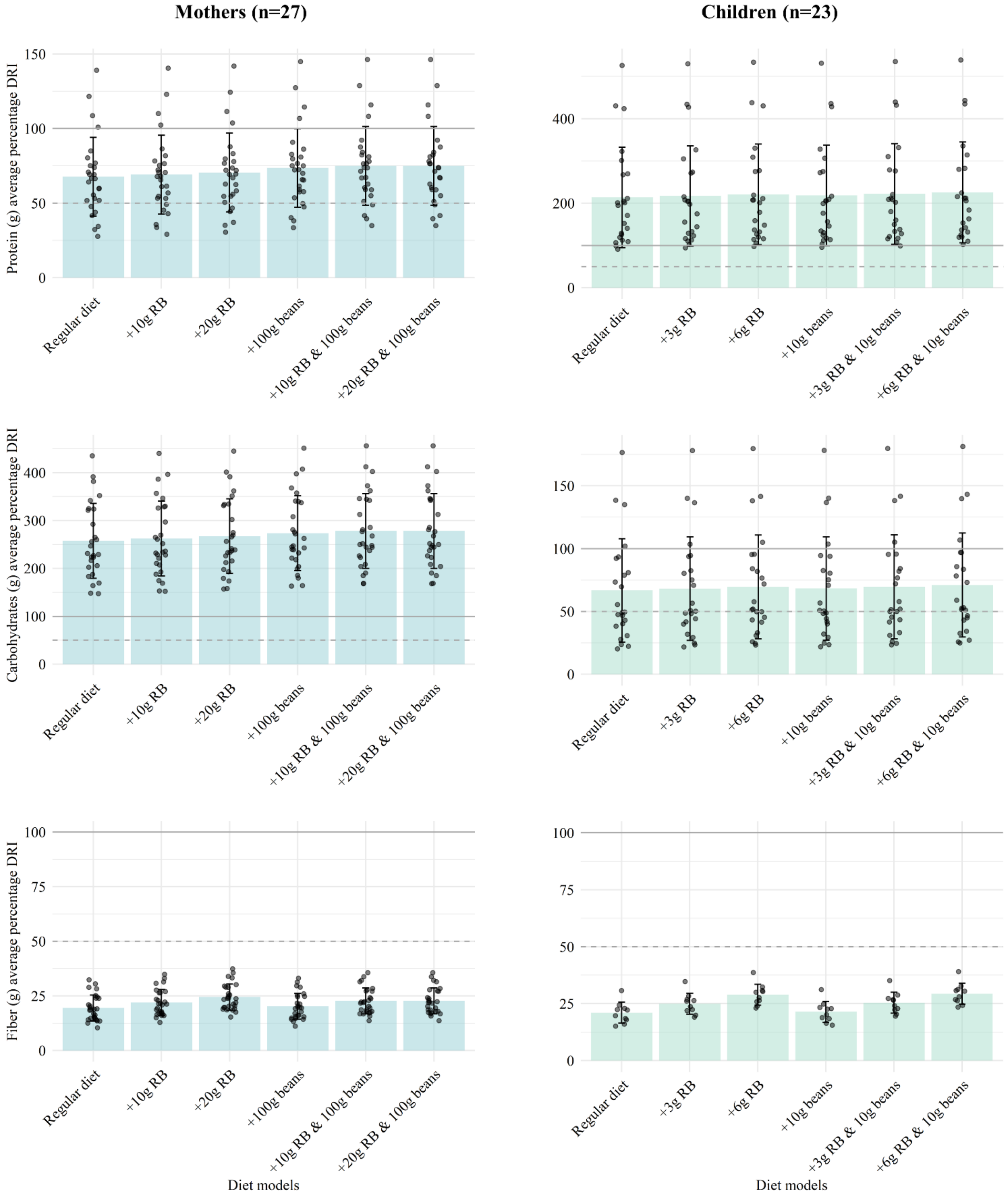

Supplemental figure S4. Maternal (n=27) and child (n=23) average percent of dietary reference intakes (DRIs) achieved for protein (g), carbohydrates (g), and fiber without dietary intervention (regular diet) and modelled with the addition of rice bran, black beans, and rice bran plus black beans. Dots represent individuals. As there is no DRI for fiber for children <12 months old, fiber values for children are for those aged 13-24 months only.

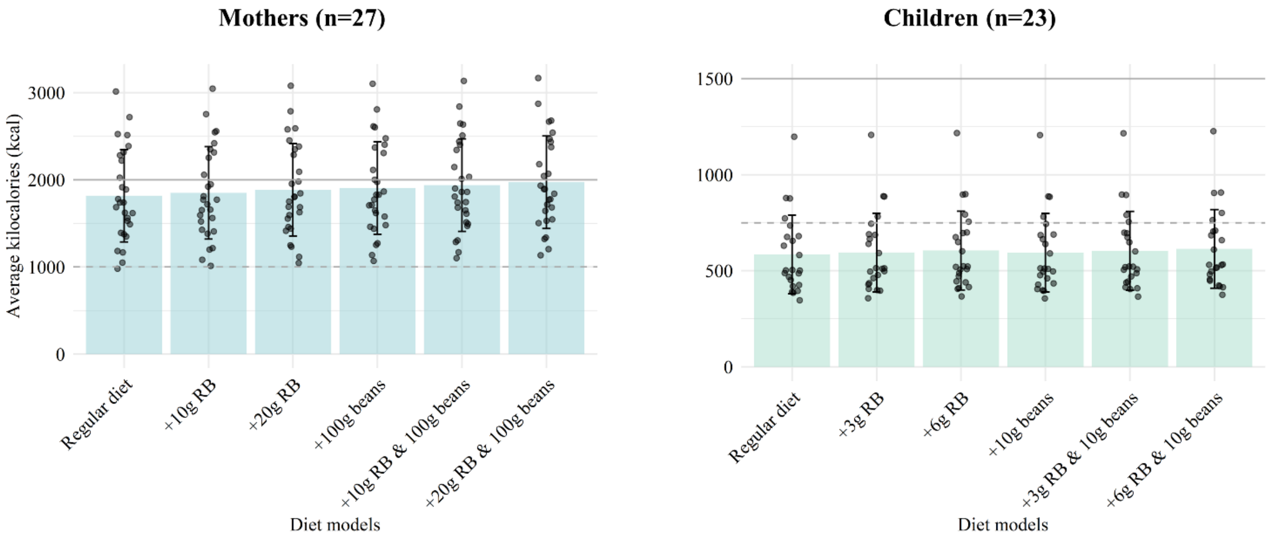

Supplemental figure S5. Maternal (n=27) and child (n=23) average kilocalorie (kcal) intake between baseline and endline dietary recalls and modelled with the addition of rice bran, black beans, and rice bran plus black beans. Dots represent individuals.

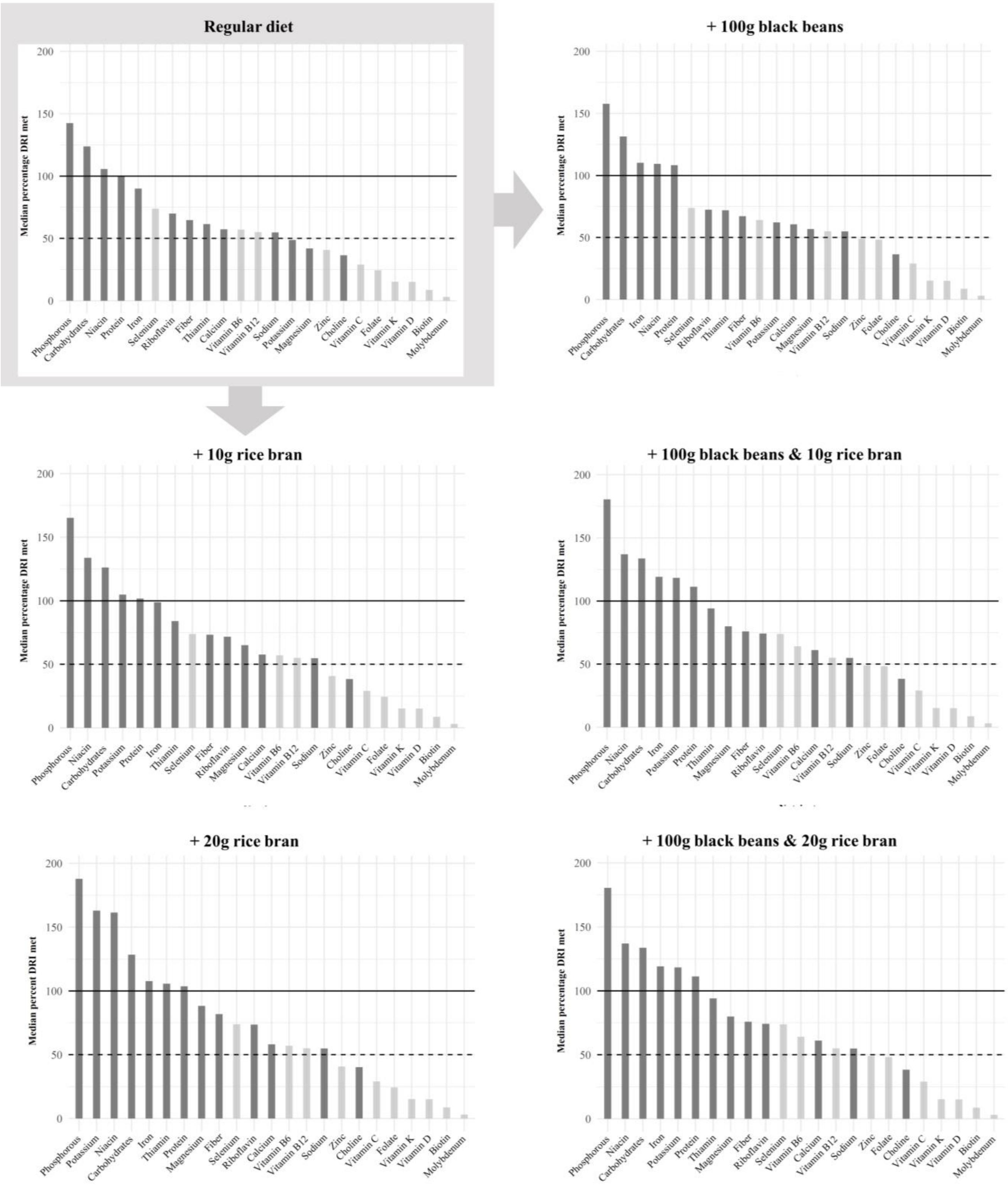

Supplemental figure S6. Maternal (n=27) median percent of the nutrient dietary reference intakes (DRIs) achieved without dietary intervention (regular diet) and modelled with the addition of rice bran (10g and 20g), black beans (100g), and rice bran with black beans (110g and 120g). Nutrients are included on the x-axis and ordered by highest to lowest percentage for each image. Bars highlighted in dark gray are nutrients to which rice bran contributes.

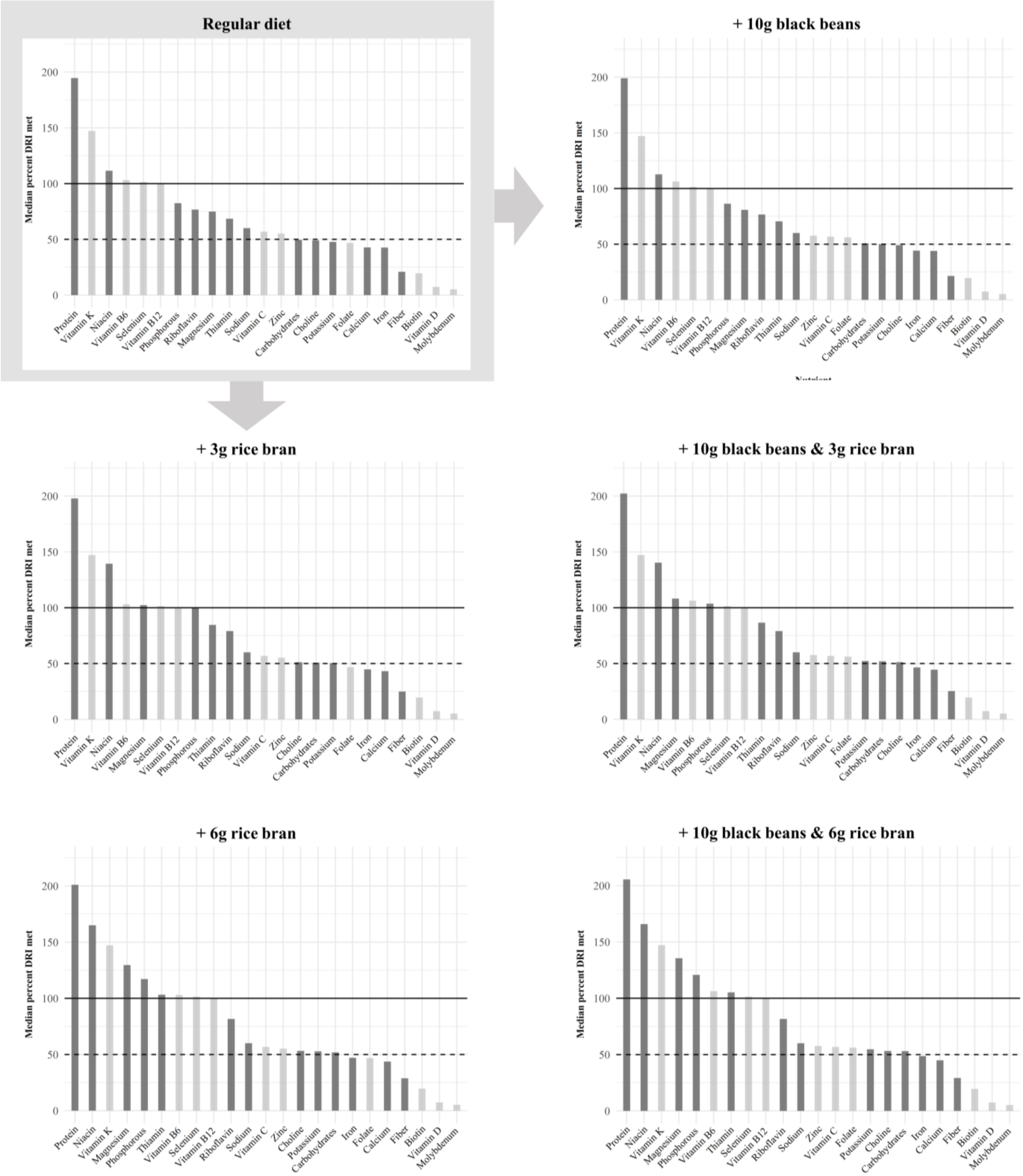

Supplemental figure S7. Child (n=23) median percent of the nutrient dietary reference intakes (DRIs) achieved without dietary intervention (regular diet) and modelled with the addition of daily rice bran (3g and 6g), black beans (10g), and rice bran with black beans (13g and 16g). Nutrients are included on the x-axis and ordered by highest to lowest percentage for each image. Bars highlighted in dark gray are nutrients to which rice bran contributes.
